# Depressurization-based reinjection method for low-permeability sandstone geothermal reservoirs

**DOI:** 10.1038/s41598-026-40426-5

**Published:** 2026-02-24

**Authors:** Mingming Lu, Zhongcheng Li, Li Chen, Ming Li, Chao Fan, Anci Wang, Xiaohong Xu, Hangzhou Xiao

**Affiliations:** Institute of Exploration and Development, PetroChina Jilin Oilfield Company, Songyuan, 138000 Jilin China

**Keywords:** low-permeability sandstone, geothermal reinjection, pressure drawdown cone, depressurization-based production, Energy science and technology, Engineering, Solid Earth sciences

## Abstract

The development of low-permeability sandstone geothermal reservoirs is constrained by high reinjection pressures associated with conventional direct reinjection. To address the lack of consideration of reservoir pressure field evolution in existing studies, this paper proposes a depressurization-based reinjection method for low-permeability sandstone geothermal reservoirs. The method adopts staged fluid production to form a pressure drawdown cone, thereby enabling low-pressure reinjection. Taking the H block of the Jilin Oilfield as a case study, a three-dimensional heterogeneous geological model was constructed to simulate reservoir pressure distributions under different depressurization flow rates, and to optimize the depressurization flow rate and well spacing. The results show that, compared with direct reinjection, when the depressurization rate is 600 m³/d and the well spacing is 250–300 m, the reinjection pressure can be reduced by more than 80%, effectively alleviating the high-pressure reinjection challenge in low-permeability sandstone reservoirs. This study provides practical design guidance for the sustainable development of geothermal resources.

## Introduction

Geothermal reinjection is a key technology for geothermal resource development. Previous studies have shown that reinjection performance is jointly influenced by wellbore structure, drilling operations, reinjection fluid quality, and multiple reservoir clogging mechanisms^1–3^. Among these factors, mechanical clogging caused by suspended particles and biological clogging induced by bacterial and microbial growth are widely recognized as the primary causes of reinjection performance degradation^4,5^. Although drilling-induced formation damage can be mitigated through optimized operations, it cannot be completely avoided^6–8^. With improvements in water treatment precision, clogging risks have been significantly reduced^9–11^; however, excessively high reinjection pressure remains a common problem.

For sandstone geothermal reservoirs, reinjection practice indicates that clogging and insufficient injectivity persist even under strict water treatment conditions. Low reservoir permeability often requires extremely high reinjection pressures, severely limiting the success of sustainable reinjection^12,13^. Recent studies have further demonstrated that sandstone reservoirs are highly sensitive to chemical precipitation, clay migration, and microbial activity^14,15^, and that injectivity is jointly controlled by pore connectivity as well as chemical and biological clogging processes^16^. Although various stimulation techniques can temporarily enhance injectivity, intrinsic low permeability and limited pressure propagation remain the fundamental constraints^17^. Therefore, water treatment and stimulation technologies alone cannot fundamentally overcome the inherent hydraulic limitations of low-permeability sandstone reservoirs.

Most existing studies have focused on optimizing wellbore design and injection conditions, while relatively few have addressed reinjection feasibility from the perspective of reservoir pressure field regulation. In this study, a depressurization-based reinjection strategy is proposed for low-permeability sandstone geothermal reservoirs. Taking the Jilin Oilfield as the study area, a production–reinjection well pair in the H block was selected as the research object. By integrating field engineering practice with numerical simulation, a three-dimensional heterogeneous geological model was constructed to analyze the influence of reservoir pressure field evolution on reinjection pressure. The proposed method was incorporated into a coupled fluid–heat transfer model and validated using field data. The results provide scientific support for geothermal reinjection in the Jilin Oilfield and offer a generalizable technical framework for efficient and sustainable reinjection in low-permeability sandstone geothermal systems.

## Overview of the Study Area

**Fig. 1:**
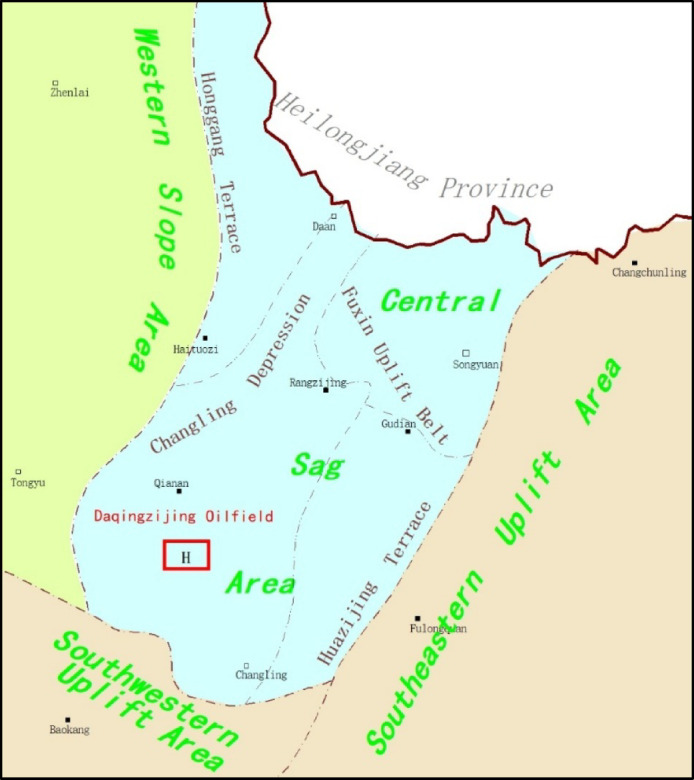
Location map of the target H block in the study area.

The Daqingzijing Oilfield is located in Daqingzijing Township, Qian’an County, Jilin Province, within the Jilin exploration area, approximately 30 km north of Qian’an County and about 70 km south of Changling County^18^. The terrain is relatively flat, with elevations ranging from 140 to 165 m, and surface conditions are favorable for geothermal development. The target H block is situated in the Changling Depression of the central sag area in the southern Songliao Basin (Fig. 1), the Qingsan Member is well developed, with aquifers buried at depths of 1,900–2,100 m and individual water-bearing layers reaching thicknesses of 30–40 m. Reservoir property data indicate that the Qingsan Member exhibits an average porosity of approximately 12.3% and an average permeability of about 0.005 μm², classifying it as a low-porosity, low-permeability sandstone reservoir. Formation temperature ranges from 68 to 75 ℃, indicating clear geothermal development potential. Detailed geological and reservoir parameters are summarized in Table 1.

The H and H-3-1 wells were selected as the primary observation wells, where four step-drawdown pumping tests were conducted. The maximum dynamic water level reached − 601 m, and the maximum stabilized production rate was 132.48 m³/d, indicating significant drawdown but limited productivity and reflecting the poor permeability of the reservoir. Following reservoir hydraulic fracturing treatment^19^, production performance improved substantially, with average production rates increasing to approximately 300 m³/d. The maximum reinjection rate reached 450 m³/d, accompanied by a reinjection pressure of 2.2 MPa, indicating that hydraulic fracturing effectively enhanced reservoir permeability and rendered reinjection technically feasible. The reinjection pressure remains relatively high due to the inherently low reservoir permeability, making this area an ideal test site for evaluating the proposed depressurization-based reinjection method in low-permeability sandstone geothermal reservoirs.

**Table 1:** Petrophysical properties of the Qingsan Member sandstone geothermal reservoir in the H block.

Study area	Aquifer depth (m)	Aquifer thickness (m)	Porosity (%)	Permeability (um^2^)	Formation temperature(℃)
H block, Qingsan Member	1900–2100	30–90	3.8–23.9	0.002–0.01	68–75

## Depressurization-based reinjection method

**Fig. 2:**
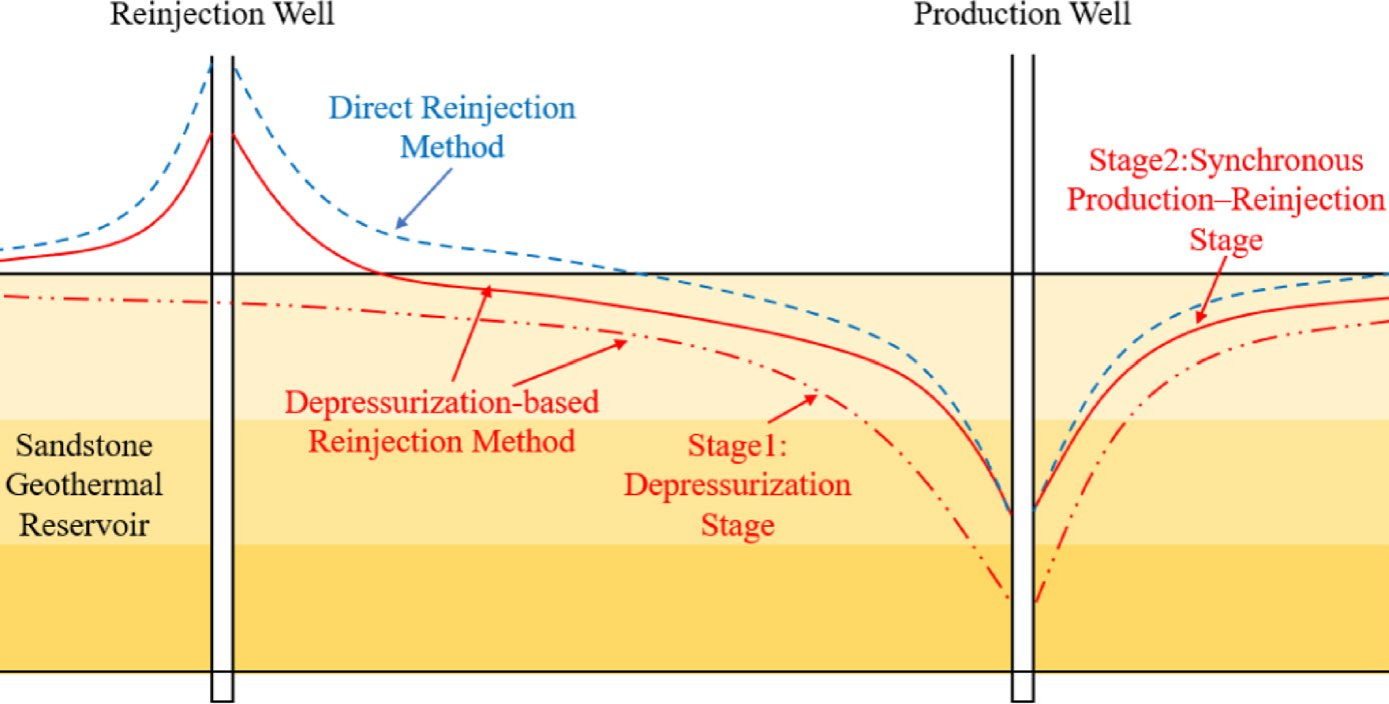
Schematic comparison between the conventional direct reinjection method and the depressurization-based reinjection method.

In contrast to the conventional direct reinjection scheme, this study proposes a staged production–reinjection strategy, in which reservoir depressurization is first induced through controlled fluid production, leading to the reservoir of a stable pressure drawdown cone. Following the depressurization stage, the operational mode is consistent with that of the direct reinjection approach, with both production and reinjection conducted at fixed flow rates (Fig. 2). On this basis, a depressurization-based reinjection method for low-permeability sandstone geothermal reservoirs is established, and production and reinjection parameters are systematically optimized to achieve the targeted depressurization-assisted reinjection performance.

### Construction of a heterogeneous geological model

Wells H and H-3-1, located in the primary development area of the H block, possess comprehensive pumping test, hydraulic fracturing, and reinjection operational data, all exhibiting large drawdown, low productivity, and high reinjection pressure, and were therefore selected as the study objects. Based on integrated well logging data, a well-scale production–reinjection model was constructed, and key reservoir parameters such as porosity and permeability for both fractured and unfractured zones were obtained from pre- and post-fracturing test data, which were subsequently used to establish a three-dimensional heterogeneous geothermal reservoir model.

### Development of a coupled flow–heat transfer model

Coupled multiphysics numerical simulations were conducted using COMSOL Multiphysics to simulate the evolution of groundwater level induced by fluid production and reinjection in the geothermal reservoir, that is, the pressure variation caused by fluid flow in the porous medium. The target reservoir fluid in the study area is a stable single-phase liquid with sufficiently low compressibility to neglect density variations due to pressure changes^20^. Accordingly, the fluid is assumed to be a single-phase incompressible liquid obeying Darcy’s law, with reservoir rock and fluid in local thermal equilibrium. Production and reinjection wells operate at constant flow rates, and the initial pressure field is defined as hydrostatic.

The fluid migration within the porous medium is described by Darcy’s law:

1 $$u= - \frac{k}{\mu }\left( {\nabla p - {\rho _f}g\nabla z} \right)$$

Where, *u* represents the Darcy velocity, m/s; *k* represents the reservoir permeability, m^2^ ; $\mu$represents the dynamic viscosity of the fluid, Pa•s; *p* represents the fluid pressure, Pa; ${\rho _f}g\nabla z$represents the gravity term, where ${\rho _f}$represents the fluid density, kg/m^3^, *g* represents the gravitational acceleration, 9.8 m/s^2^, and $\nabla z$represents the gradient of the vertical coordinate, its physical meaning is equivalent to the unit vector ${e_z}$ in the vertical direction. The fluid continuity equation is expressed as:

2 $$\varphi \frac{{\partial {\rho _f}}}{{\partial t}}+\nabla \cdot \left( {{\rho _f}u} \right)={Q_m}$$

Where, $\varphi$ represents the reservoir porosity; *t* represents time, s ;${Q_m}$represents the mass source term, accounting for fluid production or reinjection in the wellbore, kg/(m^3^•s). The heat transfer process associated with fluid seepage in the reservoir occurs via both conduction and convection, and can be described by the heat transfer equation for porous media:

3 $${(\rho {C_p})_{eff}}\frac{{\partial T}}{{\partial t}}+{\rho _f}{C_{p,f}}u\cdot \nabla T=\nabla \cdot ({\lambda _{eff}}\nabla T)+Q$$

Where, *T* represents the reservoir temperature, K ; ${C_{p,f}}$represents the specific heat capacity of the fluid, J/ ( kg • K ) ; *Q* represents the volumetric heat source term, accounting for the energy input or output induced by wellbore production or reinjection, W/m^3^; ${(\rho {C_p})_{eff}}$represents the effective volumetric heat capacity of the reservoir at atmospheric pressure, and ${\lambda _{eff}}$represents the effective thermal conductivity of the reservoir, which can be expressed as:

4 $${(\rho {C_p})_{eff}}={\theta _s}{\rho _s}{C_{p,s}}+{\theta _f}{\rho _f}{C_{p,f}}$$

5 $${\lambda _{eff}}={\theta _s}{\lambda _s}+{\theta _f}{\lambda _f}$$

Where, ${\rho _s}$represent the rock density, kg/m^3^, ${C_{p,s}}$represent the specific heat capacity of the rock, J/(kg•K), ${\theta _s}$, ${\theta _f}$represent the volume fractions of the fluid and the rock, respectively; ${\lambda _s}$, ${\lambda _f}$represent the thermal conductivities of the rock and fluid, respectively, W/(m•K).

### Design and specification of model parameters

Depressurization-based reinjection involves continuously producing fluid from production wells at a prescribed rate prior to reinjection, thereby reducing reservoir pressure and directing the reinjected fluid toward low-pressure zones to decrease reinjection resistance and enhance reinjection efficiency. Based on this concept, a staged production–reinjection strategy is proposed, with different production and reinjection parameters designed to simulate reservoir pressure distributions at each stage. It is assumed that after m years, the system enters the synchronous production–reinjection stage, during which the wells operate simultaneously, and the production and reinjection rates are defined as piecewise functions of time as follows:

6 $${q_{prod}}\left( t \right)=\begin{array}{*{20}{c}} {{q_1},0<t \leqslant m} \\ {{q_2},m<t \leqslant n} \end{array},{q_{inj}}\left( t \right)=\begin{array}{*{20}{c}} {0,0<t \leqslant m} \\ {{q_2},m<t \leqslant n} \end{array}$$

Where, *m* represents the duration of the depressurization production stage, years; n represents the total operation time, years; ${q_{prod}}\left( t \right)$ and ${q_{inj}}\left( t \right)$ represents the production rate of the production well and the injection rate of the reinjection well, respectively, m^3^/d; ${q_1}$ represents the production rate during the depressurization stage, m³/d; ${q_2}$ represents the production–injection rate during the synchronous stage, m³/d.

## Numerical simulation and discussion

### Model parameters and boundary conditions

**Fig. 3:**
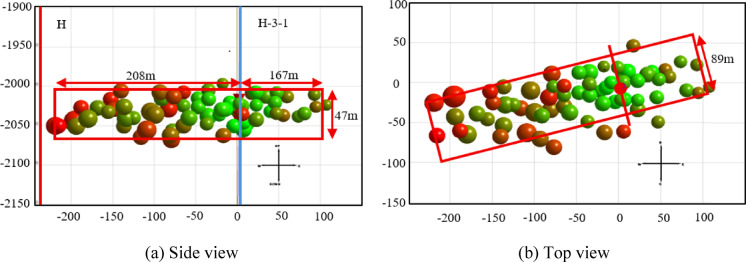
Microseismic distribution of hydraulic fracturing in well H-3-1.

To improve computational efficiency, the geological model was established only for the target reservoir. The production well H and reinjection well H-3-1 are spaced 285 m apart, with the model dimensions set to 1000 m×1000 m ×50 m. Based on the actual perforation intervals, the well lengths of H and H-3-1 are 41.5 m and 51.6 m, respectively, with a wellbore diameter of 0.1397 m. Incorporating fracture monitoring and microseismic interpretation data (Fig. 3), the fracture zone is modeled as a cuboid with dimensions of 300 m×80 m×40 m. According to single-well drawdown test fitting results before and after fracturing^19^, the unfractured zone exhibits a permeability of 0.002 μm² and a porosity of 0.15, while the fractured zone has an enhanced permeability of 0.035 μm² and porosity of 0.20.

**Fig. 4:**
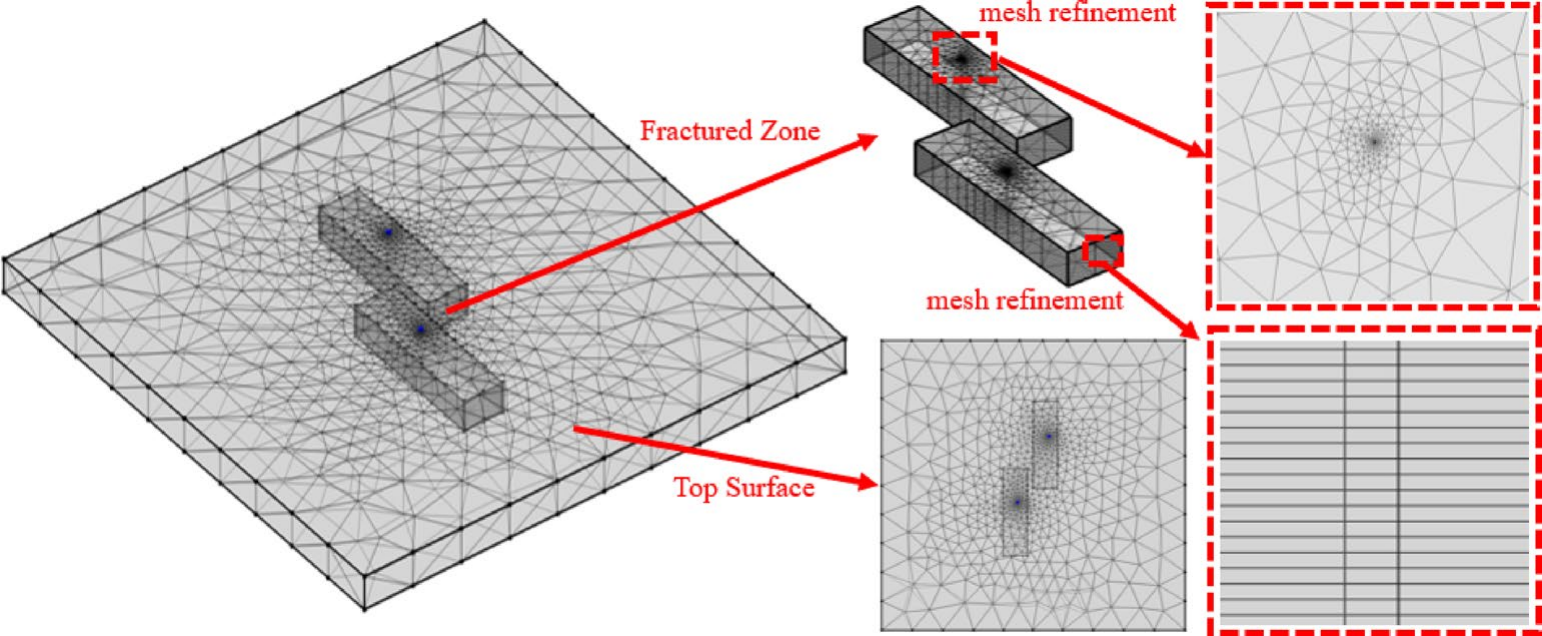
Three-dimensional heterogeneous production–reinjection well model.

The initial pressure and temperature are linearly distributed along the vertical direction, with a pressure gradient of 9810 Pa/m and a geothermal gradient of 0.031 ℃/m. The top and bottom boundaries of the model are set as no-flow and thermally insulated, while the lateral boundaries are prescribed as constant-head and open boundaries with a water head of 1 mm(Table 2). To reduce the influence of overall mesh scale on numerical simulation results and enhance computational accuracy^21–23^, the wells are simplified as one-dimensional elements, and an unstructured mesh with regional refinement is applied (Fig. 4). The top surface of the fracture zone is discretized with triangular elements, swept downward into prismatic elements, with local mesh refinement around the wells, yielding a total of 20,040 elements, with mesh sizes ranging from 0.1 m to 50 m. Simulations are solved using the Newton iterative algorithm with a time step of 10 days. Considering that this study focuses on pressure variation and reinjection performance, the adopted mesh resolution and time-step settings are sufficient to represent reservoir pressure evolution and the reinjection process while maintaining computational efficiency. Model parameters are summarized in Table 3.

**Table 2:** Boundary Conditions of Production–Reinjection Wells Model.

Boundary	Hydraulic Condition	Thermal Condition
Top	No-flow	Thermally insulated
Bottom	No-flow	Thermally insulated
Lateral	A water head of 1 mm	Open boundary

**Table 3:** Key parameters of the production–reinjection well model.

Parameter	Value
Reservoir Depth (m)	2000–2050
Initial Water Saturation	1.0
Rock Density (kg/m³)	2440
Rock Specific Heat Capacity (J/(kg·K))	920
Rock Thermal Conductivity (W/(m·K))	2.5
Porosity	0.15
Permeability (µm²)	0.002
Surface Temperature (°C)	10
Geothermal Gradient (°C)	0.031
Bottom Temperature (°C)	72
Bottom Pressure (MPa)	20
Fracture Zone Dimensions (Length × Width × Height, m)	300 × 80 × 40
Fracture Zone Porosity	0.20
Fracture Zone Permeability (µm²)	0.035
Production Well	Well H
Production Flow Rate (m³/d)	300–600
Reinjection Well	Well H-3-1
Reinjection Flow Rate (m³/d)	300–450

### Model simulation and validation

**Fig. 5:**
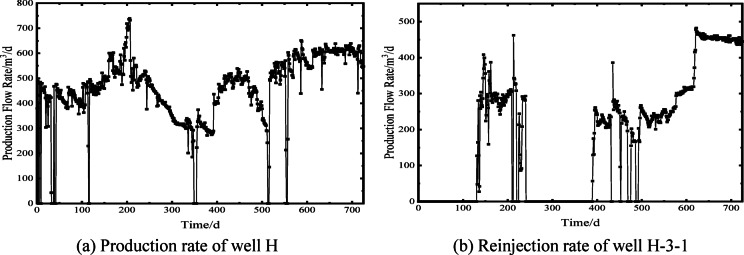
Actual operating rates of production well H and reinjection well H-3-1.

**Fig. 6:**
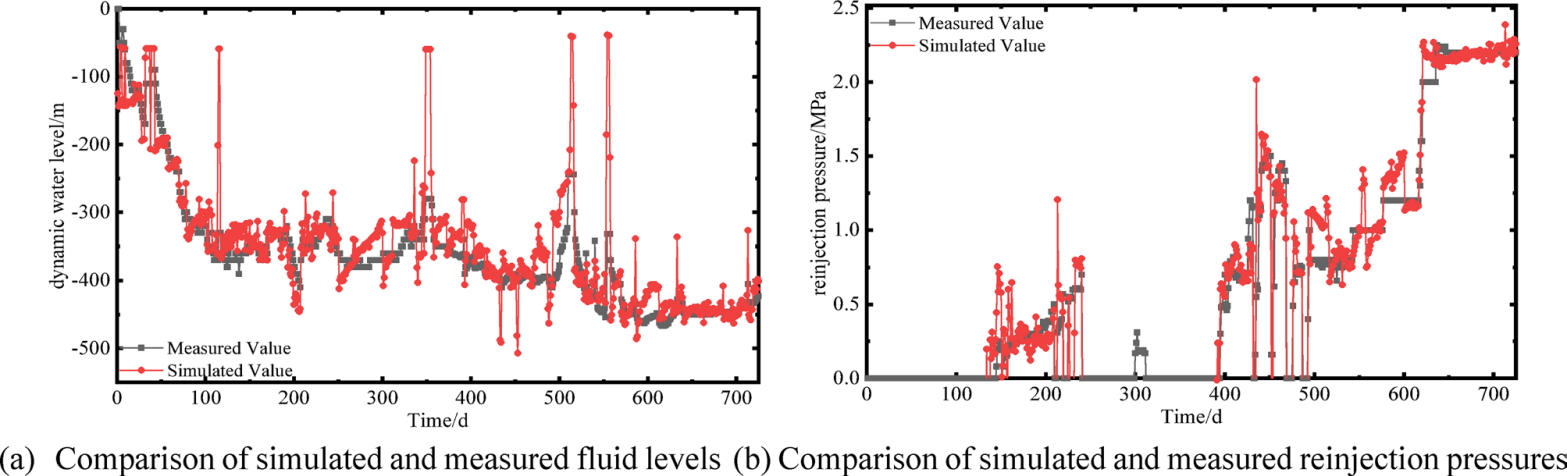
Validation of numerical model results against field monitoring data.

**Fig. 7:**
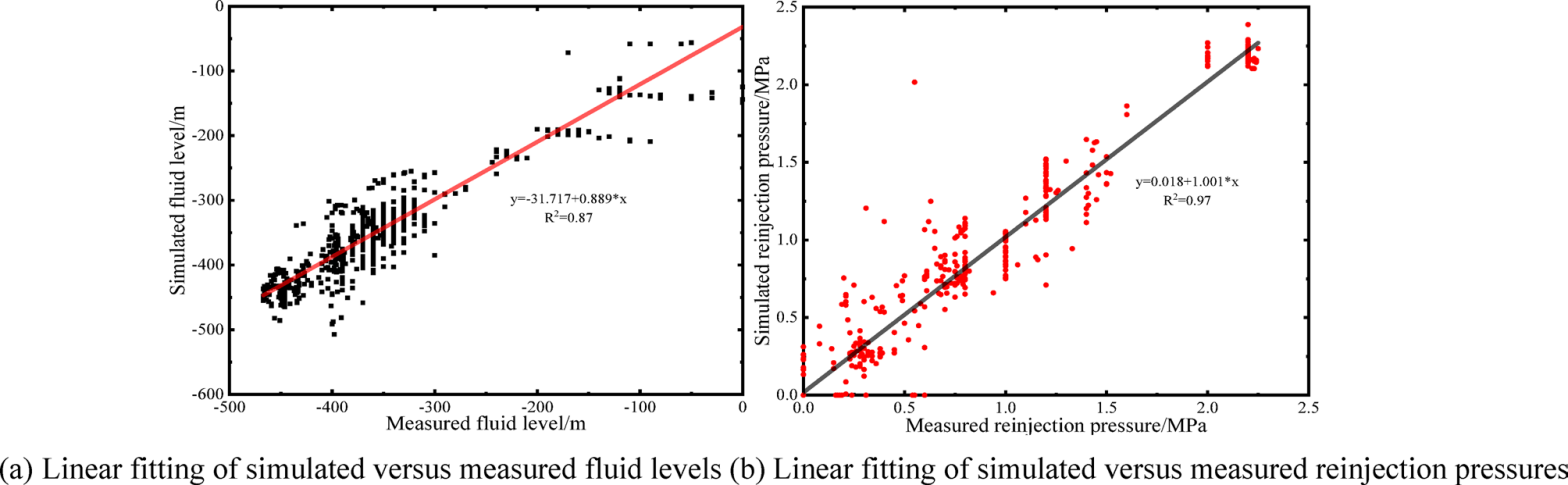
Accuracy validation of the numerical model for fluid levels and reinjection pressures.

Based on the aforementioned model parameters, mesh discretization scheme, and time-step settings, numerical simulations of the dynamic production data from February 2023 to February 2025 (Fig. 5), the pumping fluid level of well H and the reinjection pressure of well H-3-1 were simulated. The reinjection pressure is defined as the additional pressure required for reinjection relative to the initial reservoir pressure. The simulated results were validated against field monitoring data (Fig. 6), showing good agreement with measured values. The coefficients of determination R^2^ of linear fitting were 0.87 and 0.97, respectively (Fig. 7), indicating that the model accuracy is sufficient for subsequent analyses.

**Fig. 8:**
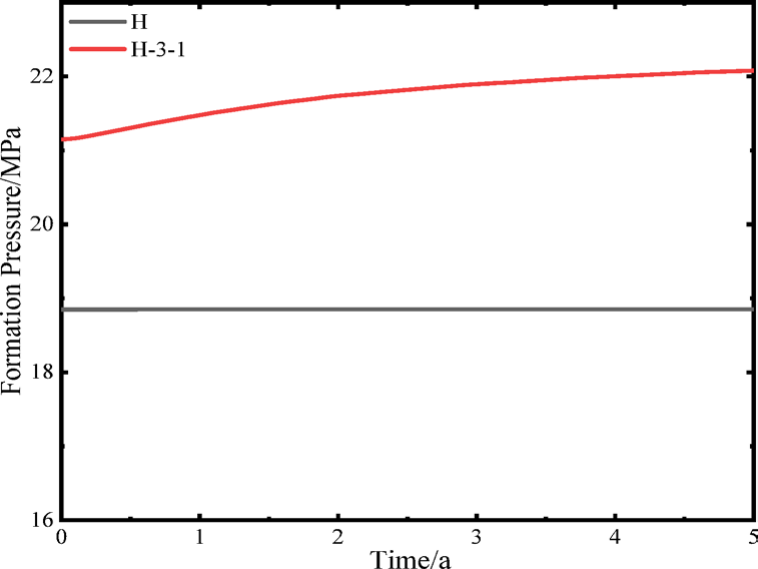
Reservoir pressure of production wells over a five-year operation period.

Since the measured data cover only two years, to avoid over-extrapolation of long-term reservoir pressure variations, the simulation period was set to five years. During this period, the direct reinjection method was applied with an average production rate of 300 m³/d to simulate reservoir pressure changes of the production wells over five years (Fig. 8). The results indicate that the pressure of well H reached 18.8 MPa, while well H-3-1 reached a maximum of 22.1 MPa, with reinjection pressures up to 2.1 MPa. These results were further compared with the depressurization-based reinjection method to evaluate the effectiveness of the proposed approach.

### Design and optimization of simulation parameters

The proposed approach divides the operational period into two stages: during the first year, only well H undergoes depressurization production while well H-3-1 is not reinjected; from the second to the fifth year, both wells operate simultaneously at equal production and reinjection rates, with a constant flow rate of 300 m³/d. On this basis, the depressurization flow rate of well H is varied at 300, 450, 600, 750, and 900 m³/d, and the corresponding production and reinjection rates of wells H and H-3-1 are as follows:

7 $${q_{prod}}\left( t \right)=\begin{array}{*{20}{c}} {300/450/600/750/900,0<t \leqslant 1} \\ {300,1<t \leqslant 5} \end{array},{q_{inj}}\left( t \right)=\begin{array}{*{20}{c}} {0,0<t \leqslant 1} \\ {300,1<t \leqslant 5} \end{array}$$

Reservoir pressure changes of both wells are calculated to predict the reinjection pressure of well H-3-1 and to optimize the depressurization flow rate of well H.

Well spacing also has a notable impact on reinjection performance. Under constant flow conditions, the spacing is varied at 150, 200, 250, 300, and 350 m, and the corresponding reservoir pressure changes and reinjection pressures are simulated to determine the optimal well spacing.

### Results and Discussion

#### Influence of Different Depressurization Flow Rates on Reinjection Pressure

**Fig. 9:**
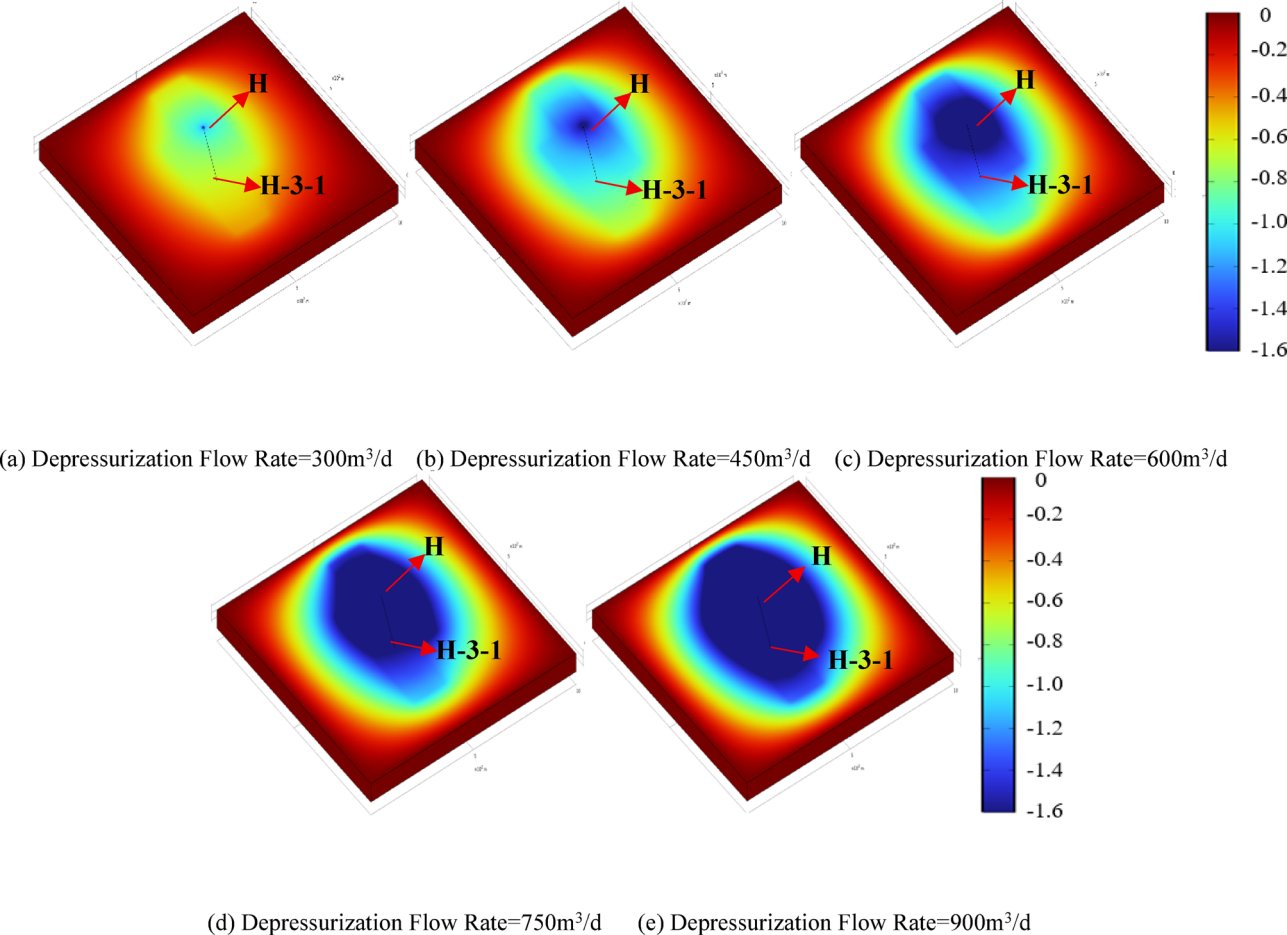
Reservoir pressure drop distribution after one-year operation under different depressurization flow rates.

**Fig. 10:**
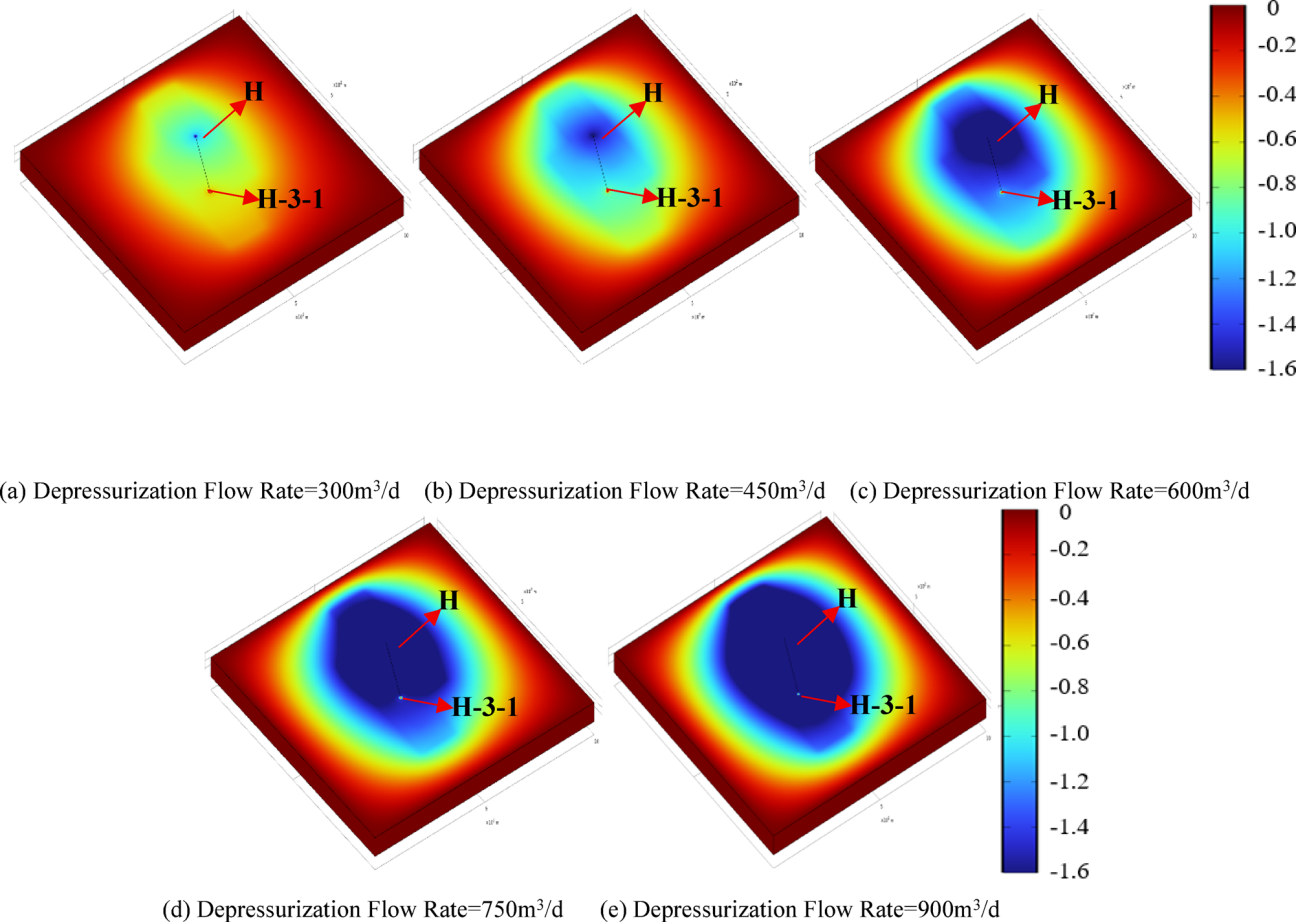
Reservoir pressure drop distribution after five-year operation under different depressurization flow rates.

**Fig. 11:**
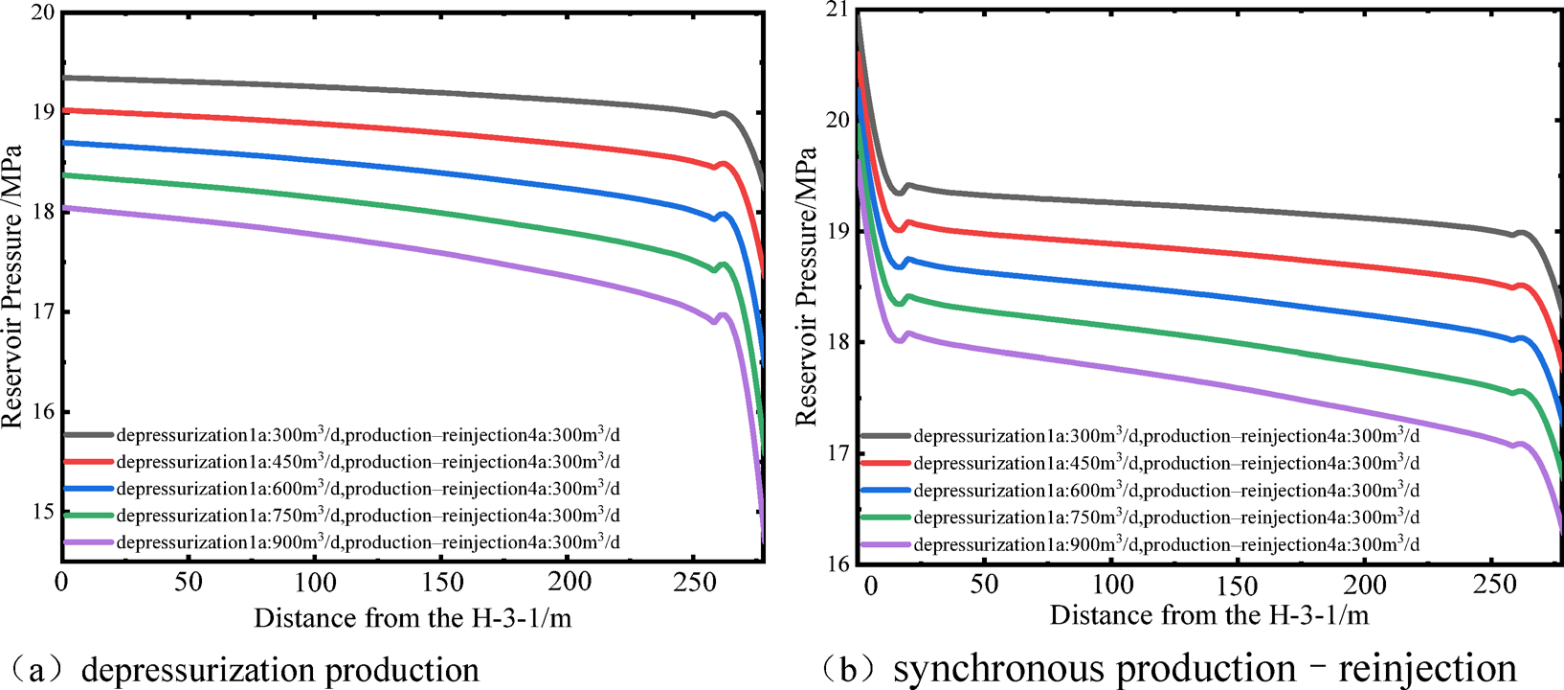
Reservoir pressure distribution between wells H and H-3-1 under different depressurization flow rates.

Figures 9 and 10 present the reservoir pressure distribution around wells H and H-3-1 after the two-stage operation, where colors from red to blue represent high to low pressure. The results indicate that the depressurization production forms an elliptical pressure drawdown cone along the production–reinjection direction. At low depressurization flow rates, the pressure drawdown cone is mainly concentrated near well H, and the reservoir pressure around well H-3-1 fully recovers after reinjection. As the depressurization flow rate increases, the pressure drawdown cone gradually extends toward well H-3-1, resulting in reservoir pressures below the initial value after reinjection, indicating a more pronounced pressure gradient at higher flow rates. Figure 11 shows the reservoir pressure distribution between the two wells, with the horizontal axis representing the distance along the line connecting well H and H-3-1. The low-permeability reservoir at the far end limits pressure propagation, and local low-pressure and high-pressure zones form within approximately 20 m around each well. The reservoir pressure distribution is primarily controlled by the production and reinjection rates: higher depressurization flow rates release more pressure relative to the initial 20 MPa reservoir pressure, leading to steeper pressure gradients between the wells. At the same production or reinjection rate, the near-well pressure gradients around well H and H-3-1 are generally consistent, indicating that flow rate is the dominant factor controlling inter-well pressure distribution and reinjection pressure variations.

**Fig. 12:**
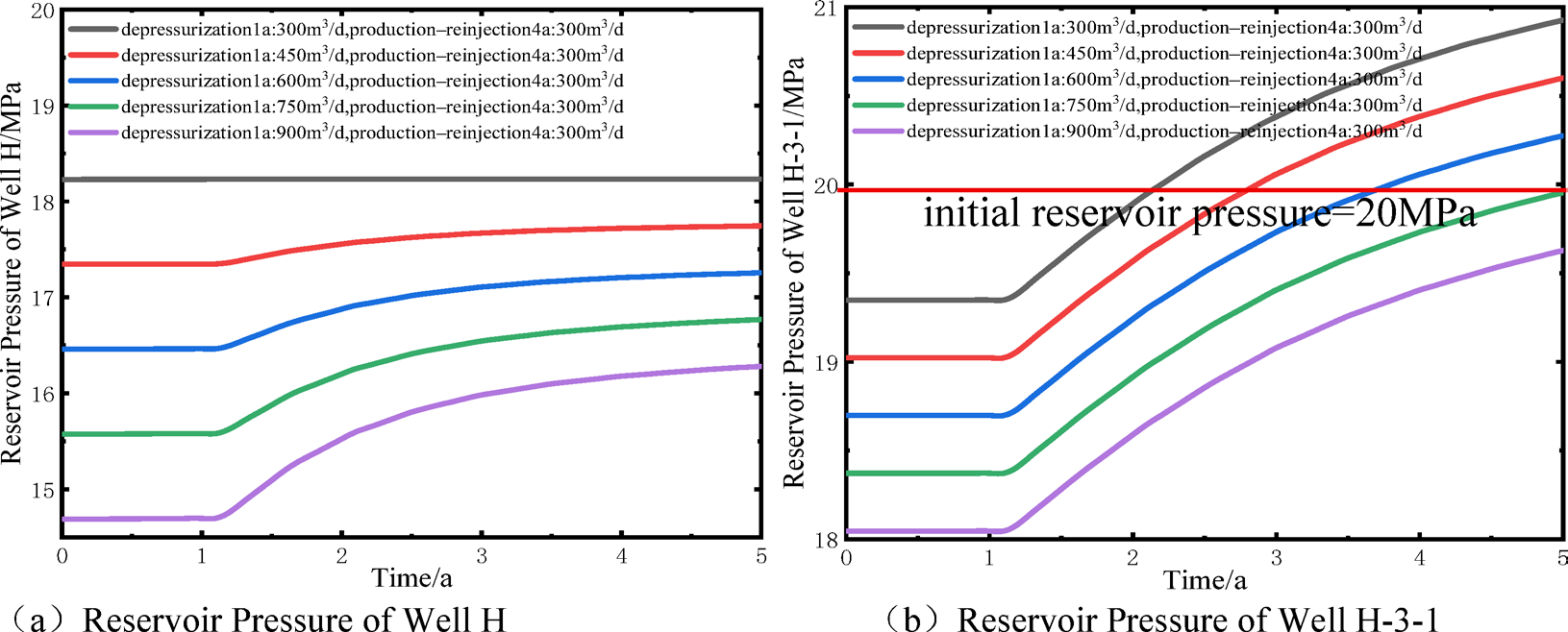
Bottom-hole reservoir pressure distribution of wells H and H-3-1 under different depressurization flow rates.

Figure 12 further quantifies the impact of different depressurization flow rates on well pressure changes and reinjection performance. During the depressurization production stage, each 150 m³/d increase in flow rate reduces the pressures of wells H and H-3-1 by 0.88 MPa and 0.33 MPa, respectively. During the subsequent synchronous production–reinjection stage, the same flow rate increment reduces the pressures of the two wells by 0.49 MPa and 0.33 MPa, respectively. After reinjection, the pressure recovery rate of well H is approximately 56% of the depressurization-induced pressure drop, while the overall pressure of well H-3-1 increases by about 1.58 MPa, demonstrating that reinjection effectively mitigates the pressure differences caused by varying depressurization flow rates. Based on these results, the reinjection pressure of well H-3-1 was calculated. After 1.1, 1.6, and 2.6 years of reinjection, the maximum reinjection pressures under depressurization flow rates of 300 m³/d, 450 m³/d, and 600 m³/d are 0.9 MPa, 0.6 MPa, and 0.3 MPa, respectively; at 750 and 900 m³/d, no additional pressure is required. During the depressurization production stage, well H pressures decrease by 9%, 13%, 17%, 22%, and 26% relative to the initial reservoir pressure. Excessive pressure drop may induce near-well compaction and reduced permeability^24^, based on the operational conditions of the Jilin Oilfield, a maximum pressure reduction of 20% is recommended, corresponding to a depressurization flow rate of 600 m³/d as the optimal scheme. Compared with the direct reinjection method, the proposed approach reduces the reinjection pressure by 85.7%, significantly improving reinjection conditions and lowering operational risks. Rational depressurization production is therefore a key measure to enhance reinjection efficiency and optimize the reservoir pressure field.

#### Influence of well spacing on reinjection pressure

**Fig. 13:**
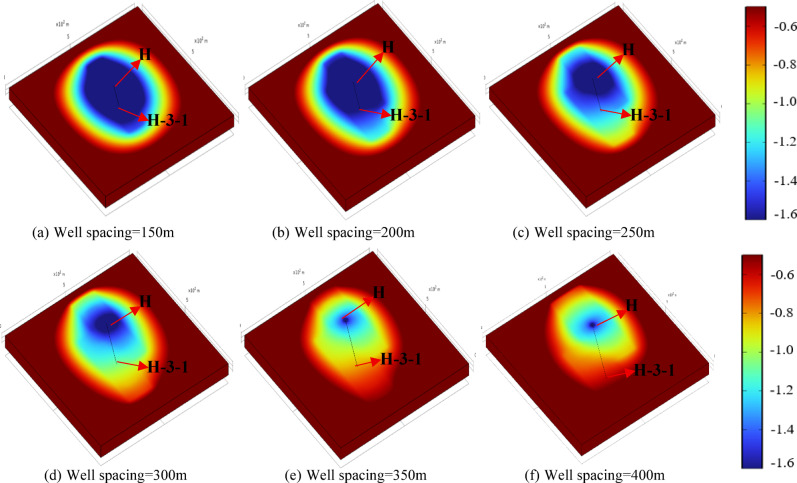
Reservoir pressure drawdown distribution after 1 year of operation under different well spacings.

**Fig. 14:**
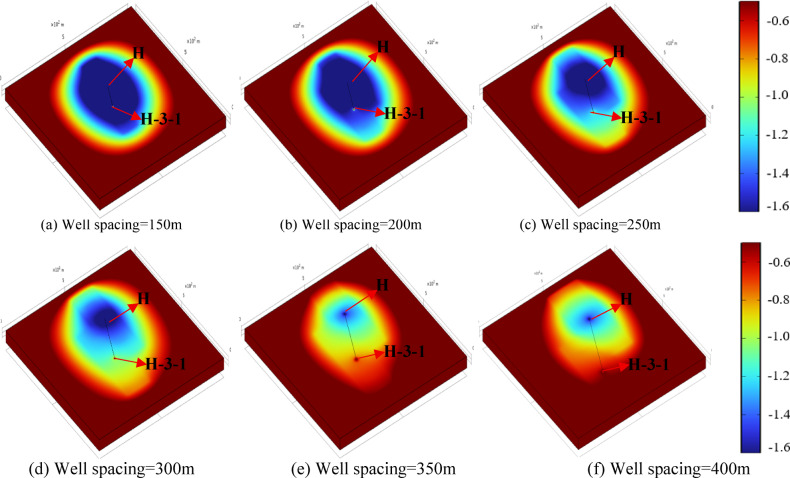
Reservoir pressure drawdown distribution after 5 years of operation under different well spacings.

Well spacing exerts a certain influence on fluid flow and reinjection pressure. Under a depressurization flow rate of 600 m³/d and a synchronous production–reinjection flow rate of 300 m³/d, the reservoir pressure variations of wells H and H-3-1 were analyzed. Figures 13 and 14 present the pressure distributions during the depressurization production and synchronous production–reinjection stages, respectively. The results indicate that when the well spacing is small, the pressure drawdown cone formed by well H extends fully to well H-3-1, resulting in only partial pressure recovery after reinjection. As the spacing increases, the pressure drawdown cone covers most of the H-3-1 well region, allowing greater pressure recovery after reinjection. At large well spacings, H-3-1 is minimally affected by depressurization, and its pressure almost fully returns to the initial level, indicating that well spacing primarily affects inter-well pressure coupling, while overall pressure recovery remains dominated by the production–reinjection flow rate.

Table 4 compares reservoir pressures under depressurization-based reinjection and direct reinjection at different well spacings. Under the direct reinjection method, the pressure of well H is approximately 18.8 MPa, and the reinjection pressure of well H-3-1 is about 2.1 MPa, demonstrating that in low-permeability sandstone reservoirs, well spacing has a limited effect on reinjection pressure. As the well spacing increases, reservoir pressures under the depressurization-based reinjection method shows a gradual increasing trend. When the spacing exceeds 350 m, the two wells are almost unaffected by each other, and no significant production–reinjection coupling is observed. Compared to the depressurization production stage, reinjection increases pressures of wells H and H-3-1 by approximately 0.8 MPa and 1.5 MPa, respectively, further confirming that pressure recovery is mainly controlled by the flow rate, with minor influence from well spacing. Relative to direct reinjection, the depressurization-based reinjection method reduces pressure by over 10% at its maximum, effectively alleviating pre-reinjection pressure loads.

**Table 4:** Reservoir pressures of wells H and H-3-1 under different well spacings.

Well	Reservoir pressure at well H					Reservoir pressure at well H-3-1				
Method	Depressurization-based reinjection			Direct reinjection		Depressurization-based reinjection			Direct reinjection	
Well spacing (m)	Depressurization production (MPa)	Synchronous production–reinjection (MPa)	After reinjection (MPa)	Pressure (MPa)	Bottom-hole pressure reduction(%)	Depressurization production (MPa)	Synchronous production–reinjection (MPa)	After reinjection (MPa)	Pressure (MPa)	Bottom-hole pressure reduction(%)
150	15.91	16.74	0.83	18.90	12.9	18.07	19.70	1.63	22.08	10.8
200	16.17	16.98	0.81	18.89	11.2	18.39	19.93	1.54	22.04	9.6
250	16.46	17.26	0.80	18.88	9.4	18.70	After 3.8a, max 20.28	1.58	22.12	8.3
300	16.64	17.45	0.81	18.85	8.0	18.93	After 3.3a, max 20.41	1.48	22.02	7.3
350	16.97	17.79	0.82	18.89	6.2	19.2	After 2.4a, max 20.79	1.59	22.18	6.3
400	16.92	17.71	0.79	18.77	6.0	19.38	After 2.1a, max 20.91	1.53	22.06	5.2

Simulations of H-3-1 reinjection pressures over a five-year operation period show that when well spacing is within 200 m, no additional pressure is required. For spacings exceeding 200 m, the maximum reinjection pressures after 2.8, 2.3, 1.4, and 1.1 years are 0.3 MPa, 0.4 MPa, 0.8 MPa, and 0.9 MPa, respectively. During the depressurization production, the pressure of well H decreases by 20.5%, 19.2%, 17.7%, 16.8%, 15.2%, and 15.4% relative to the initial formation pressure; for spacings below 200 m, the pressure reduction approaches or exceeds 20%, which may induce near-well compaction and permeability loss^24^. Considering reinjection efficiency and engineering feasibility, the optimal well spacing is determined to be 250–300 m. Within this range, reinjection pressure is reduced by more than 80% compared to the direct reinjection method, achieving efficient reinjection while optimizing inter-well layout.

#### Evaluation of heat extraction and thermal breakthrough

**Fig. 15:**
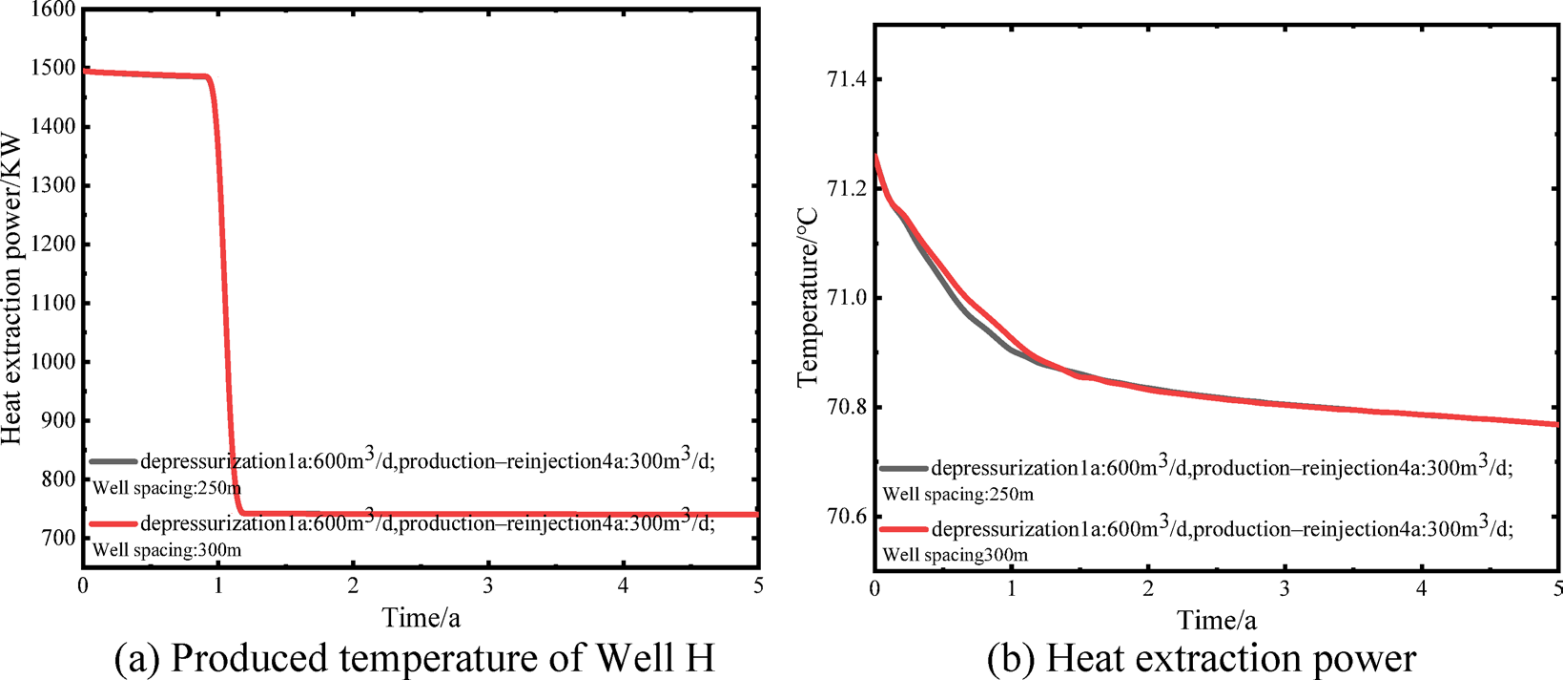
Produced temperature of Well H and system heat extraction power during 5 years of operation.

To ensure consistency between the pressure and temperature fields during production and reinjection, a coupled flow–heat transfer model was employed. Based on the optimized depressurization flow rate and well spacing, the variation in production temperature at well H was analyzed to evaluate potential thermal breakthrough risks. Figure 15(a) shows the production temperature of well H over a 5-year operation period. At a depressurization flow rate of 600 m³/d and a well spacing of 250 m, the temperature decreases from 71.26 ℃ to 70.90 ℃, while at a spacing of 300 m it decreases to 70.93 ℃, indicating that increasing well spacing slightly mitigates temperature decline. After reinjection at a rate of 300 m³/d, the temperature further decreases to 70.77 ℃ for both cases; however, the rate of temperature decline is significantly reduced compared with the depressurization production stage. Overall, the production temperature of well H remains stable under both well spacings, with a total decline of less than 0.5 ℃ and no evidence of thermal breakthrough during the simulation period. After reinjection, the production temperatures tend to converge.

The primary objective of the initial depressurization production stage is reservoir pressure regulation, while simultaneously quantifying the thermal energy extracted by the produced fluid. Figure 15(b) illustrates the system heat extraction power over the 5-year period, using 20 ℃ as the reference temperature. During the depressurization production stage, the heat extraction power is approximately 1495 kW for both well spacings. After reinjection, as production rates decrease and the temperature field gradually stabilizes, the heat extraction power declines to approximately 742 kW. These results indicate the magnitude of recoverable thermal energy in the produced fluid, which can be effectively managed using a closed-loop heat exchange system. This provides thermal support for subsequent reinjection operations, facilitating production–reinjection balance and demonstrating the feasibility of conventional geothermal development under the proposed scheme.

## Conclusions

This study proposes a depressurization-based reinjection method for low-permeability sandstone geothermal reservoirs. Through a combination of numerical simulations and engineering practice, the feasibility and advantages of forming a pressure drawdown cone prior to reinjection were systematically evaluated. The results demonstrate that moderate depressurization production can establish an effective pressure drawdown cone covering the reinjection well, thereby significantly reducing reinjection pressure and improving reinjection efficiency. Well spacing primarily controls the hydraulic connectivity between production and reinjection wells, and an optimal spacing of 250–300 m achieves a balance between reinjection performance and inter-well coupling effects. Under optimized conditions, low-pressure or near-zero-pressure reinjection can be achieved, with reinjection pressures substantially lower than those required by conventional direct reinjection methods. This improvement effectively enhances reinjection feasibility in low-permeability reservoirs, increases the sustainability of geothermal development, and reduces operational energy consumption.

Furthermore, the proposed approach provides a quantitative design reference for optimizing production rates, well spacing, and reinjection schemes in low-permeability sandstone geothermal reservoirs. It should be noted that the impacts of long-term thermal extraction and reservoir heterogeneity on pressure field evolution and reinjection performance were not fully considered in this study. Future work may integrate real-time monitoring data and reservoir heterogeneity characteristics to further optimize operational strategies, thereby better accommodating field conditions and guiding well pattern design and reservoir management.

## Data Availability

The datasets used and/or analysed during the current study available from the corresponding author on reasonable request.
